# Iterative guided machine learning-assisted systematic literature reviews: a diabetes case study

**DOI:** 10.1186/s13643-021-01640-6

**Published:** 2021-04-02

**Authors:** John Zimmerman, Robin E. Soler, James Lavinder, Sarah Murphy, Charisma Atkins, LaShonda Hulbert, Richard Lusk, Boon Peng Ng

**Affiliations:** 1 Deloitte Consulting, LLP, 191 Peachtree Street, Atlanta, GA 30303 USA; 2 Centers for Disease Control and Prevention, National Center for Chronic Disease Prevention and Health Promotion, Division of Diabetes Translation, 1600 Clifton Rd, Atlanta, GA USA; 3 College of Nursing & Disability, Aging and Technology Cluster, University of Central Florida, 12201 Research Pkwy Suite 300, Orlando, FL USA

**Keywords:** Machine learning, Systematic review screening, Natural language processing, Transfer learning, Machine learning configurations, Applied case study

## Abstract

**Background:**

Systematic Reviews (SR), studies of studies, use a formal process to evaluate the quality of scientific literature and determine ensuing effectiveness from qualifying articles to establish consensus findings around a hypothesis. Their value is increasing as the conduct and publication of research and evaluation has expanded and the process of identifying key insights becomes more time consuming. Text analytics and machine learning (ML) techniques may help overcome this problem of scale while still maintaining the level of rigor expected of SRs.

**Methods:**

In this article, we discuss an approach that uses existing examples of SRs to build and test a method for assisting the SR title and abstract pre-screening by reducing the initial pool of potential articles down to articles that meet inclusion criteria. Our approach differs from previous approaches to using ML as a SR tool in that it incorporates ML configurations guided by previously conducted SRs, and human confirmation on ML predictions of relevant articles during multiple iterative reviews on smaller tranches of citations. We applied the tailored method to a new SR review effort to validate performance.

**Results:**

The case study test of the approach proved a sensitivity (recall) in finding relevant articles during down selection that may rival many traditional processes and show ability to overcome most type II errors. The study achieved a sensitivity of 99.5% (213 out of 214) of total relevant articles while only conducting a human review of 31% of total articles available for review.

**Conclusions:**

We believe this iterative method can help overcome bias in initial ML model training by having humans reinforce ML models with new and relevant information, and is an applied step towards transfer learning for ML in SR.

**Supplementary Information:**

The online version contains supplementary material available at 10.1186/s13643-021-01640-6.

## Background

Systematic reviews (SR), studies of studies, use a formal process to evaluate the quality of scientific literature and determine ensuing effectiveness from qualifying articles to establish consensus findings around a hypothesis. Because SRs involve pooling information, conducting a SR can result in increased power and reduced bias in determining effectiveness, increased generalizability of findings, and can help identify publication trends, research gaps, and other indicators of importance [1]. Their value is increasing as the conduct and publication of research and evaluation has expanded and the process of identifying key insights becomes more time consuming [2]. Text analytics and machine learning (ML) techniques may help overcome this problem of scale while still maintaining the level of rigor expected of SRs.

Where SR methods historically depend on human resources for each stage of the process. ML is a computer-based technique that uses statistics and pattern recognition to create models to make predictions from data to automate processes. One area of SRs that has seen application of ML is pre-screening title and abstracts for final inclusion in the quality scoring step [3]. In this article, we discuss an approach that uses existing examples of SRs to build and test a method for assisting the SR title and abstract pre-screening by reducing the initial pool of potential articles down to articles that meet inclusion criteria. This iterative and guided method is aimed at maintaining sensitivity in finding relevant articles during pre-screening, while reducing the number of articles reviewed compared to traditional SR methods. We incorporate many of the features of other ML approaches for SRs to our approach including reinforcing training data with human reviews of select articles, using only a small number (< 200) of articles for initial ML training, and feature engineering of data for improved performance in selecting articles [4–9]. Our approach differs from previous approaches to using ML as a SR tool in that it incorporates testing the proposed process on previously conducted SRs to guide ML parameters and configurations, and attempts to optimize the process of human confirmation on ML predictions of relevant articles during multiple iterative reviews by selecting only articles that will ensure sensitivity is reached. We also incorporate a quality checkpoint and initial determination of when to stop iterations of ML in the process to build confidence that a desired sensitivity has been reached without trusting only the ML effort or reviewing all articles. This guided method is also meant to be portable to new SR topics, and not just the ones that configurations were built upon. To test this, we used our guided configurations on a new SR need as a case study to see how our guided configurations perform on a previously untested topic.

## Methods

Common features of the SR process include developing a research question, developing inclusion criteria, conducting a literature search, article title and abstract pre-screening, abstracting articles for analysis, and aggregating results to generate summary findings [2]. Our ML application efforts focused on creating efficiency in article title and abstract pre-screening. We refer to this step as “down selection” - reducing the initial pool of potential articles that meet inclusion criteria. To develop a generalizable ML approach to down selection, we (1) created a theoretical ML “down selection” process based on goals and constraints of including ML in a down selection process, (2) established ML configuration guides by testing settings with experiment SRs, and (3) applied the process and ML configuration guides to a SR conducted by a team of scientists at the Centers for Disease Control and Prevention (CDC).

### Developing a theoretical ML “down selection” process

**Fig. 1:**
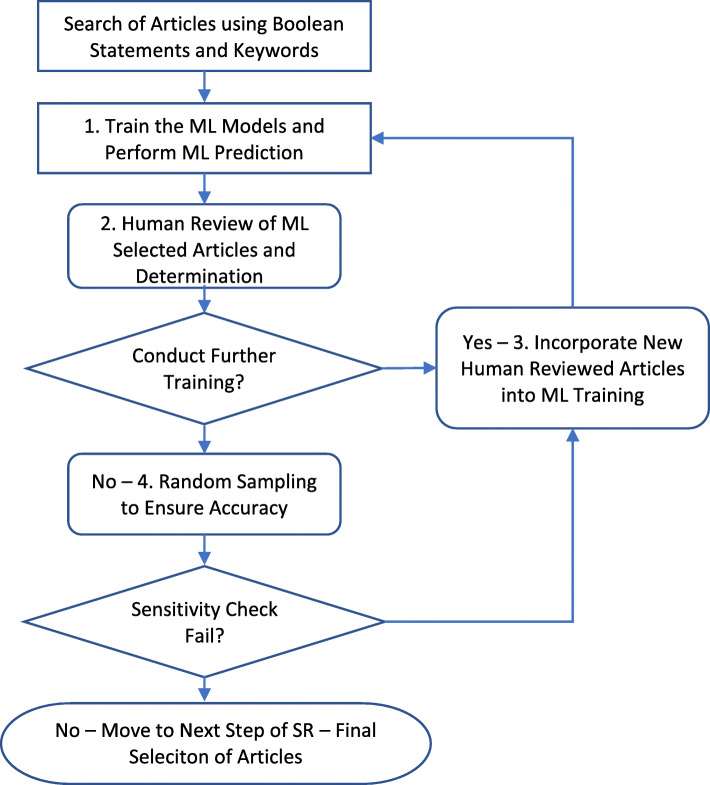
Flow chart of proposed approach to operationalize machine learning in a down selection process

To operationalize the ML addition to the down selection process [2], we developed a process based on the constraints of ML and SRs. The steps in the proposed process include (1) train ML models and perform ML predictions; (2) conduct human review of articles selected by the ML model and determine accuracy of prediction; (3) incorporate new human reviewed articles into iterative ML training; (4) random sampling to ensure performance (Fig. 1).

#### Step 1—train the ML models and perform ML prediction

Supervised ML algorithms require training data to build models. These data “teach” the algorithms which articles meet inclusion criteria, and which do not, facilitating the creation of a model [10, 11]. Our process aimed to show that effective training data can come from a small set of articles that are pre-identified as meeting or not meeting inclusion criteria. In addition, a small volume (less than 200) random sampling of articles can be drawn from a keyword literature search or through unsupervised machine learning [7]. Iterative training will occur later in the proposed process after more articles are reviewed to reinforce learning.

In our proposed process, a ML model makes predictions, after training, on combined title and abstract text review. Through this process, each potential article fed to the model is given a score of how likely it is to fit inclusion criteria based on articles in the training data set. Prediction scores are probabilistic ranging from 0 to 1.0, with 1.0 being a perfect match to inclusion examples [12].

#### Step 2—human review of ML selected articles and determination

In this step, human reviewers examine articles predicted by the ML model to fit inclusion criteria. During review, humans correct the ML prediction by confirming if an article met inclusion criteria or did not. From this process a new training set (human reviewed) is created for use in a new iteration of training. This iterative training process is proposed to help improve ML prediction by expanding training sets and overcoming potential bias introduced by small training sets in step 1.

#### Step 3—incorporate new human reviewed articles into iterative ML training

For each iteration, a new model is trained on the set of human reviewed articles in step 2 and any previous training sets. The iterated model is then employed on the remaining unreviewed articles to determine a new predictive score. Iterations should continue until the number of articles predicted as relevant becomes small and human review does not confirm articles predicted to be relevant.

#### Step 4—random sampling to ensure accuracy

After exiting step 3, humans select a random sample of articles not predicted as relevant by ML to test sensitivity of the ML process. For our process we suggested a 99% confidence level sample with a 10% margin of error for calculating the total articles for random sampling to ensure confidence. We recommend this process to increase confidence that all inclusion articles have been identified. Humans check this random selection of articles to look for articles that fit review criteria. If more than one or two articles are found that fit inclusion criteria, this would indicate the ML approach has not reached a reasonable sensitivity and should continue for a new iteration (step 2).

### Establish ML configurations for the down selection process

Supervised ML has a superabundant number of configurations for predictive model development. Areas identified that could have multiple configurations include (a) cleaning text; (b) reducing dimensionality; (c) feature engineering; (d) developing a training sample; (e) initial algorithm selection and assessment; (f) creating a soft voting stacked model; and (g) choosing thresholds for the iterative modeling steps. To identify which ML configurations should be utilized for the proposed ML down selection process we utilized a hybrid theoretical and results-driven approach by testing on four previously completed SRs hereby referred to as experiment SRs. Configurations for steps a, b, c, and d were selected based on theoretical knowledge, while configurations for steps e, f, and g were selected by testing performance of different configurations for each experiment SR individually.

#### Step a—cleaning text

From each of the experiment SRs, we performed standard text cleaning on the combined titles and abstracts [13], removing numbers and common English words, and tokenizing words into single and bi-grams. We used Python’s Natural Language Toolkit (NLTK) version 3.2.4 for this process.

#### Step b—reducing dimensionality

Because our text cleaning process resulted in a data set with a large number of rows and columns (a high-dimensional matrix) that represent the numerical frequency of token occurrences, we performed dimensionality reduction [14]. We also manipulated the cleaned data into a term frequency–inverse document frequency (TF-IDF) matrix [15]. TF-IDF is a statistical weight, meant to show importance of a word is to a document and the entire series of documents in an analysis.

#### Step c—feature engineering

In ML applications, variables for modeling are often referred to as “features” of the data. Feature engineering involves manipulating variables to create new “features” of the data and is often used to boost predictive performance [16]. We utilized latent Dirichlet allocation (LDA) on the reduced TF-IDF matrix to create new features based on topics found in the data using a generative probabilistic approach [17]. Using topics instead of just word counts as features creates the ability to identify patterns across articles that does not rely on word token occurrences. We set the LDA topics at 30 new features under the theoretical assumption that 30 would reach topic saturation in the data. We also used truncated singular value decomposition (TSVD) to perform feature decomposition—reducing a matrix to its constituent parts—on the TF-IDF Matrix. This resulted in a condensed TF-IDF matrix containing the 50 most significant features in terms of their representation of the original data [18]. We also know that a literature search will typically have few articles returned that will meet inclusion criteria (imbalanced data). To address this issue, we applied a Synthetic Minority Over-sampling Technique (SMOTE), which creates new feature points aimed at overcoming imbalanced data [19]. We appended the features derived from the LDA, TSVD, and SMOTE to the reduced TF-IDF matrix to get our final matrix for ML modeling.

#### Step d—developing training sample

Our approach to creating a training set was to mimic the operational approach we outlined in the proposed ML “down-selection” process. We assumed that a small number of articles would be available for training; no more than 60 with examples from both relevant and non-relevant articles. Through this, we created our initial training data through stratified random selection of the experiment SRs articles, our test data set was the unreviewed data from experiment SRs.

#### Step e—initial algorithm selection and assessment

Many ML algorithms for building models exist. We tested the following algorithms for overall accuracy from the initial training set: support vector machine (SVM) with stochastic gradient descent, K-nearest neighbors (KNN), decision tree binary, SVM with a sigmoidal kernel, gradient boosting classifier, random forest classifier, and multinomial naive Bayes [20–23]. Python’s scikit-learn library and the base parameters of these modes were used for implementing models. From these, we chose the four models that performed the best in terms of accuracy (ratio of number of correct predictions to the total number predictions made) to include in a stacked ensemble model [24, 25], which combines the strengths of the best performing models. They were SVM with Stochastic Gradient Decent, KNN, Decision Trees, and Sigmoidal SVM.

#### Step f—compiling into a stacked ensemble model

We used a soft voting ensemble classifier to build predictive models to overcome any weakness in each individual model [25]. In a soft voting ensemble, different models are given weights that are applied to their prediction and combined for a final stacked prediction. We evaluated various weight distributions according to their area under receiver operating characteristic (ROC) curves from initial model training (step d) [26]. The [10–SVM, 1–KNN, 1–Decision Tree, 1–Sigmoidal SVM] weighting distribution consistently resulted in the highest area under the curve of the options tested. As with individual models, the stacked ensemble model predicts a value for each article from 0 to 1.0, where values closer to 1.0 indicate an article being relevant for inclusion.

#### Step g—choosing prediction thresholds for iterative modeling

Prediction thresholds in our scores (0–1.0) can be changed to influence the selection of volume of articles for review in iterations (SR down selection step 3) [27]. High thresholds result in lower volumes and vice versa. By comparing actual results of experiment SR data with different prediction thresholds, we were able to identify predictive threshold guides to optimize the selection of articles for review during iterations. Once we determined an optimal threshold for an iteration, we tested multiple thresholds on the next iteration to confirm sensitivity. We were able to accomplish this because we had known results and could simulate a human review (SR down selection step 3). Based on testing of experiment SRs, we found that three iterations, including the original training round, would reach the optimal trade off in sensitivity versus percent of articles reviewed based on a 98% sensitivity goal of finding relevant articles.

From our predictive threshold testing, we used the weighted average of best thresholds from each experiment SR as a guide for non-experimental application. These thresholds are shown in Table 1. These thresholds should be thought of as guides. Volume of articles selected for review from different thresholds should also be considered when selecting which threshold to proceed with.

**Table 1:** Average post hoc model performance

Average post hoc model performance						
Data set	Prediction threshold for 1st iteration	Prediction threshold for 2nd iteration	Prediction threshold for 3rd iteration	Total articles	% of total human-reviewed articles needed to return 95% relevant articles	% of total human-reviewed articles needed to return 98% relevant articles
1st SR review	50.0%	20.0%	20%	14,655	19.3%	24%
2nd SR review	50.1%	30.3%	44%	15,234	18.9%	25%
3rd SR review	75.0%	20.0%	20%	7,670	10.0%	34%
4th SR review	70.0%	27.5%	19.5%	1,820	30.0%	41.8%
Weighted average	57.6%	26.0%	29.5%	N/A	20.9%	29.8%

Using these ML configurations, we examined the percent of total articles needed to reach 95% and 98% sensitivity of what human reviewers selected for inclusion in the experiment SRs. On average only 21% of articles would have to be reviewed to find 95% of what the human SR selected, while 30% would have to be reviewed to find 98%, including initial training articles (Table 1).

### Case study: applying the process and ML configuration guides

In May of 2018, CDC’s Division of Diabetes Translation initiated a SR designed to describe the effectiveness of incentives in increasing enrollment and retention in chronic disease prevention and management programs.

To initiate our case study, we started with the articles identified in the CDC SR team’s literature search phase. A total of 3137 articles were returned from the literature search (following deduplication across searched databases). We applied the ML down selection process described above to this same set of 3137 articles. Four team members participated in the iterative review of ML selected articles and their inclusion in the SR. Two team members independently reviewed each assigned abstract. If there were any conflicts, they were discussed and resolved. Once the team completed the ML down selection process, unreviewed article titles were scanned to determine if our case produced acceptable results when compared to a traditional approach.

## Results

Table 2 shows a breakdown of the articles reviewed during the ML assisted process and how they compared to totals after final quality checks.

**Table 2:** Results for each iteration and random sample

	Relevant training sample (iteration)	Non-relevant sample (iteration)	Threshold selected (iteration)	Articles for review (iteration)	Relevant articles (iteration)	Non-relevant articles (iteration)	Total articles reviewed (cumulative)	Total articles not reviewed (cumulative)
First iteration	15	40	0.4	458	155	303	513	2624
Second iteration	170	343	0.3	260	43	217	773	2364
Third iteration	213	560	0.3	45	0	45	818	2319
Fourth iteration	213	605	Not selected	N/A	N/A	N/A	818	2319
Random sample	N/A	N/A	N/A	156	1	155	974	2163

### SR ML down selection step 1

To develop the first iteration of our case study, the CDC SR team identified a training set of 15 articles that met inclusion criteria, and 40 that did not. We built the trained models using the ML configuration approach identified from the experiment SRs.

### SR ML down selection step 2

We examined different prediction thresholds after the first iteration model predictions. We selected a threshold of 0.4, which resulted in 458 articles as priority for review. Although lower than the prediction threshold identified from experiment SRs, the guide threshold resulted in a number of articles thought to be too low (250) to produce enough of a robust new training set. For more information on threshold selection of each iteration, please view the supplemental material in Table S1. From the 458 articles, human review identified 155 as relevant and 303 as not relevant. We added this new set of human reviewed articles to the initial training articles for the second iteration of model training and predictions.

### SR ML down selection step 3

We examined different prediction thresholds after the second iteration predictions (Table S1). We selected a threshold of 0.3 based on experiment SR guides and number of articles which identified 260 articles as a priority for review. Human review identified 43 as relevant and 217 as non-relevant. We added this new set of human-reviewed articles to the existing training set for the third iteration of model training and predictions.

#### Results of third iteration

We examined different prediction thresholds after the third iteration predictions (Table S1). We selected a threshold of 0.3 based on experiment SR guides and number of articles, which resulted in 45 articles as priority for review. Human review identified no relevant articles. We added this new set of 45 human-reviewed articles to the existing training set for the fourth iteration of model training and predictions.

#### Results of fourth iteration

During experiment SR testing we found three iterations to reach an acceptable level of sensitivity performance. During our case, we ran a fourth iteration to understand if the newly trained model selected a large number of articles for review. After reviewing potential inclusion article volume at different prediction thresholds, we decided to move on to step 4—reviewing a random sample for an error check due to small articles volumes at low thresholds.

### SR ML down selection step 4

After three training iterations, we identified 2319 citations as non-relevant. To test for saturation in sensitivity, we randomly selected 6.7% (155 citations) of these articles for human abstract review. This created a 10% margin of error with a 99% confidence level. Results from the random sample review returned 154 articles not meeting inclusion criteria and 1 potential article that met inclusion criteria, which was subsequently identified as background material and excluded.

As a final sensitivity verification after the ML process, we conducted a human review of only the titles of all 2172 unreviewed articles. Of these, we identified six articles for abstract review. We determined these were not relevant, which aligned with the ML process predictions.

Using recognized machine learning performance statistics to evaluate our approach, we achieved a sensitivity of 99.5% (213 out of 214) of total relevant articles while only conducting a human review of 31% of total articles returned from the search (Table 3). Sensitivity of the model was paramount to model acceptability, as a missed relevant article could jeopardize consensus findings of a full review of results. These results are consistent with experiment SR results in terms of sensitivity and percent of articles reviewed to reach that sensitivity.

**Table 3:** Final results after quality check

	Number of articles	Percent of total *N* (3137)
Total articles reviewed during ML down selection process	974	31.0%
Total articles not reviewed	2163	69.0%
Total relevant articles meeting inclusion criteria during ML down selection process ML–iterative review only	213	6.8%
Total relevant articles meeting review after random sampling error check and iterative review	214	6.8%

## Discussion and limitations

Our case study was conducted on a topic with a certain volume of articles for down selection. The question of efficiency for smaller and larger volume reviews remains unanswered. ML approaches require not only the expertise needed for normal SR down selection, but also the time of persons familiar with the ML process. In addition, between each iteration, a brief period is needed for training and applying the ML models. Thus, time saved by ML exclusion of irrelevant articles may be lost while carrying out technical processes. For a low-volume down selection, the potential risk of not predicting correctly with the ML process may not be justifiable given the skillset and model training time needed. However, it is likely that the benefits outweigh the risks for high-volume reviews.

To develop our approach, we trained the model using data from four completed experiment SRs, each to answer different questions. This only provided guides for certain configurations as seen by us choosing different thresholds during our case study. Our approach could be fine-tuned using a larger number of completed review and expanding potential ML options.

Machine learning focused on prediction is a constantly advancing field through ongoing commercial and academic investments in Artificial Intelligence (AI) research and application. At the time of our research, our method was focused on decision tree, linear discriminate, and proximity approaches to prediction. Convolution neural networks start to bring to life theoretical ideas about human processing of information and are increasingly being deployed to solve complex problems such as computer vision. However, the process of ML for prediction in a down-selection process may not require advanced convolution algorithms or see an improvement in performance from them. More testing is needed to determine if deep-learning techniques would improve performance in ML assisted down-selection prediction.

Ongoing advances in the area of natural language processing (NLP) may offer improvement for ML support for systematic reviews. At the time of our research, our methods started to approach incorporating conceptual topics in text through LDA; however, a transformer method will likely improve ML support for systematic reviews in terms of accuracy as they have outperformed on common text analytics and NLP benchmark tasks compared to n-gram based methods [28]. Transformers and word-embeddings are becoming common usage at the time of writing and are bringing to life theoretical language interpretation to analyze text for conceptual understand and may feature bi-directional or spatial relationships as learning components.

In the future, it is also possible that the ML-assisted down-selection approach could be conducted not just on title and abstract data, but on full-text data. However, many literature search engine capabilities and copyright restrictions only allow for a title and abstract review to be conducted without extra monetary costs. In addition, it will only become easier to apply more advanced approaches in ML and NLP as many initiatives on AI have a mission to opensource spread new best-in-class models or allow the public to use complex NLP models instead of developing it themselves.

## Conclusions

In this paper, we described the creation and testing of an approach to use guided ML to support SRs. To our knowledge, our case study is the first test of ML-supported SR that incorporates ML guides from previous reviews and multiple iterative human reviews. This approach to using guides represents some basic steps towards transfer learning approaches for ML in systematic reviews. The case study achieved sensitivity in finding relevant articles that rivals that of traditional SR. We believe this iterative method can help overcome bias in initial model training by having humans reinforce models with new information, however ensuring multiple reviewers may still be necessary to overcome human bias in the process. We also think this iterative approach is applicable to real-world scenarios where large initial training sets are not likely to be found. Others can look to our ML configurations as guides, but more experiments can be done to fine-tune configurations and apply the latest techniques in ML and NLP.

## Supplementary Information

**Additional file 1: Table S1.** Article prediction threshold and articles to be reviewed by humans after each iteration

**Additional file 2: Table S2.** Search terms used

## Data Availability

The datasets used and/or analyzed during the current study are available from the corresponding author on reasonable request.

## Abbreviations

AI: Artificial intelligence
CDC: Centers for disease control and prevention
KNN: K nearest neighbors
LDA: Latent dirichlet allocation
ML: Machine learning
NLP: Natural language processing
ROC: Receiver operator curve
SMOTE: Synthetic minority over-sampling technique
SR: Systematic reviews
SVM: Support vector machine
TF-IDF: Term frequency–inverse document frequency
TSVD: Truncated singular value decomposition
