# Supplementary material for: Iterative guided machine learning-assisted systematic literature reviews: a diabetes case study
Source: Syst Rev. 2021 Apr 2;10:97. doi: 10.1186/s13643-021-01640-6 (PMC8017891; doi:10.1186/s13643-021-01640-6)
Supplement: Supplementary file 1 — Additional file 1: Table S1. Article prediction threshold and articles to be reviewed by humans after each iteration [file 13643_2021_1640_MOESM1_ESM.docx]

Table S1. Article Prediction Threshold and Articles to be Reviewed by Humans after Each Iteration

| Prediction Threshold | First Iteration Articles to be Reviewed | Second Iteration Articles to be Reviewed | Third Iteration Articles to be Reviewed | Fourth Iteration Articles to be Reviewed (not selected) |
| --- | --- | --- | --- | --- |
| 0.2 | 1,266 | 975 | 449 | 400 |
| 0.25 | 837 | 458 | 131 | 94 |
| 0.3 | 647 | **260 (Selected)** | **45**  **(Selected)** | 19 |
| 0.35 | 528 | 195 | 20 | 5 |
| **0.4** | **458 (Selected)** | 129 | 4 | 0 |
| 0.5 | 311 | 79 | 0 | 0 |
| 0.6 | 207 | 44 | 0 | 0 |
| 0.7 | 144 | 17 | 0 | 0 |
| 0.8 | 99 | 8 | 0 | 0 |
