# Supplementary material for: Iterative guided machine learning-assisted systematic literature reviews: a diabetes case study
Source: Syst Rev. 2021 Apr 2;10:97. doi: 10.1186/s13643-021-01640-6 (PMC8017891; doi:10.1186/s13643-021-01640-6)
Supplement: Supplementary file 2 — Additional file 2: Table S2. Search terms used [file 13643_2021_1640_MOESM2_ESM.docx]

Table S2: Search Terms Used

*Indicates wild card search

| Incentive-Related Terms | Program-Related Terms | Disease-Related Terms | Excluded Terms (Title) | Excluded Terms  (Abstract, Keywords, or Title) |
| --- | --- | --- | --- | --- |
| incentiv* | management | diabetes | cancer | child* |
| payment | weight loss | pre-diabetes | infectious disease* | animal* |
| award* | health service | diabetic | smoking | rat |
| prize* | health promotion | chronic disease | smoke | rats |
| discount* | education | high blood pressure | smoker* | mouse |
| voucher* | prevention | obesity | tobacco | mice |
| free food | program | weight | nicotine | adolescent* |
| reimburse | intervention | bmi |  | weight loss surgery |
| reimbursement | rehabilitation | glucose |  | gastric bypass |
| cash | life-style change | a1c |  | lapband |
| behavioral economics | lifestyle change | cholesterol |  | weight loss medication* |
| contingency management | behavior change | hypertension |  | orlistat |
|  | maint* | prediabetes |  | alli |
|  | adopt* | pre diabetes |  | teen* |
|  | nutrition | obese |  | bupropion |
|  | promot* | weight loss |  |  |
|  | test* | physical activity |  |  |
|  | participat* | exercise |  |  |
|  | follow-up | overweight |  |  |
|  | followup | diet |  |  |
|  | follow up | target |  |  |
|  | track* | walk |  |  |
|  | life style change | walking |  |  |
|  | register | steps |  |  |
|  | registration | nutrition |  |  |
|  | signup |  |  |  |
|  | sign-up |  |  |  |
|  | sign up |  |  |  |
|  | enroll* |  |  |  |
|  | retention |  |  |  |
|  | retain |  |  |  |
|  | encourage |  |  |  |
|  | achieve* |  |  |  |
|  | adhere* |  |  |  |
|  | wellness |  |  |  |
|  | employer |  |  |  |
|  | employee |  |  |  |
